# Supplementary material for: Modelling transmission of Middle East respiratory syndrome coronavirus in camel populations and the potential impact of animal vaccination
Source: Nat Commun. 2025 Aug 18;16:7679. doi: 10.1038/s41467-025-62365-x (PMC12361541; doi:10.1038/s41467-025-62365-x)
Supplement: Supplementary file 1 — Supplementary Information [file 41467_2025_62365_MOESM1_ESM.pdf]

Modelling transmission of MERS-CoV in dromedary camel populations and the potential impact of animal vaccination

Amy Dighe[1,2], Thibaut Jombart[1], Neil Ferguson[1]

1. MRC Centre for Global Infectious Disease Analysis, Jameel Institute, School of Public Health, Imperial College London
2. Johns Hopkins Bloomberg School of Public Health, Baltimore, USA

Supplementary material

Estimating the FoI 2
1. Study inclusion in the catalytic modelling analysis 2
2. Catalytic model schematic 2
3. Catalytic model solutions 3
4. Catalytic modelling - reparameterisation of the beta-binomial distribution 6
5. Catalytic model selection 7
6. Leave One Out (LOO) analysis of catalytic model selection and parameter estimation 9

Transmission modelling and simulating vaccine impact 13
1. Selection of R_0 values to take forward into dynamic modelling simulations 13
2. Metapopulation structure and modelling external contributions of FoI 13
3. Vaccine impact 16
   a. Incidence in older animals following vaccination of young calves 16
   b. Optimal age for reducing overall incidence – sensitivity analysis under different efficacy scenarios 17
   c. Impact of vaccination by coverage under different efficacy scenarios 18

References 20

Estimating the FoI

1. Study inclusion in the catalytic modelling analysis

In total, three studies that were included in the systematic review of MERS-CoV seroprevalence in dromedaries[1] were excluded from our analysis. Two of these studies were excluded as their sampling strategies were not appropriate for FoI estimates which assumes a random cross-sectional sample of seroprevalence. The first sampled dromedaries with epidemiological connection to human cases[2], and the second sampled camels in response to finding a dromedary which tested positive for MERS-CoV during what turned out to be the height of an outbreak[3]. Finally, the third study was excluded because it was not possible to determine whether the sample population[4] overlapped with another larger study conducted in the same area that was included[5]. To make the geographical range of the FoI estimates as comprehensive as possible, seroprevalence measures from one additional study published after the systematic review were also included. This further study included measures of seroprevalence in camel populations in Senegal and Uganda - two additional countries not represented in the literature previously[6].

2. Catalytic model schematic

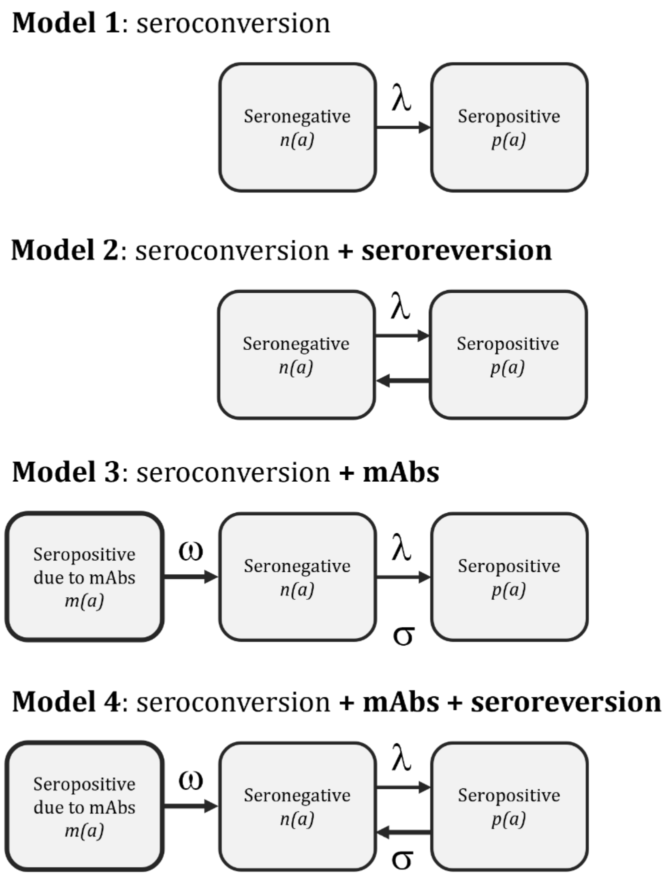

***Figure S1**. Four catalytic models of MERS-CoV seroconversion in dromedary populations, where λ is the force of infection, ω is the rate of waning of mAbs, and σ is the rate of waning of antibodies following infection.*

3. Catalytic model solutions

In Model 1, all animals are initially seronegative, becoming irreversibly seropositive at rate λ (the FoI) as has been widely applied to infectious disease prevalence data previously[7]:

$$p(a) = 1 - e^{-\lambda a} \quad \text{S1}$$

Where $p(a)$ is the expected proportion of animals that are seropositive at age a.

Model 2 (formally described by ***Equation S2***) allows for possible seroreversion - the waning of protective antibodies acquired through natural infection at rate *s*. Formulation of Model 3 was informed by catalytic models of seroconversion described and used to estimate the FoI of dengue by Imai *et al.*[8].

$$p(a) = \frac{\lambda}{\lambda + \sigma}\left(1 - e^{-(\lambda+\sigma)a}\right) \quad \text{S2}$$

We then considered the possible contribution of mAbs in Model 3 as described formally in ***Equation S3*** where m_0 is the proportion of calves born with mAbs, which then wane at rate w. We estimated m_0 by solving the equivalent model in the absence of mAbs to find the proportion of adults of reproductive age that are predicted to be seropositive and therefore pass their antibodies onto their calves.

$$p(a) = 1 - e^{-\lambda a} - m_0\left(\frac{\lambda}{\lambda - \omega}\left(e^{-\omega a} - e^{-\lambda a}\right)\right) \quad \text{S3}$$

Model 4 brings mAbs and seroreversion together (as described by ***Equation S4***).

$$p(a) = \frac{\lambda}{\lambda + \sigma}\left(1 - e^{-(\lambda+\sigma)a}\right) - m_0 \frac{\lambda}{\lambda + \sigma - \omega}\left(e^{-\omega a} - e^{-(\lambda+\sigma)a}\right) \quad \text{S4}$$

Noting that $\mu e^{-\mu(a-4)}$ is the age distribution of animals over 4 years of age, m_0 is given by

$$m_0 = \int_4^\infty \mu e^{-\mu(a-4)} p(a)da$$

$$= \int_4^a \frac{\lambda\mu e^{4\mu}}{\lambda + \sigma}\left(e^{-\mu a} - e^{-(\lambda+\sigma+\mu)a}\right) - m_0 \frac{\lambda\mu e^{4\mu}}{\lambda + \sigma - \omega}\left(e^{-(\omega+\mu)a} - e^{-(\lambda+\sigma+\mu)a}\right) da$$

$$= \frac{\lambda}{\lambda + \sigma}\left[1 - \frac{\mu}{\lambda + \sigma + \mu} e^{-4(\lambda+\sigma)}\right] - m_0 \frac{\lambda\mu}{\lambda + \sigma - \omega}\left[\frac{e^{-4\omega}}{\omega + \mu} - \frac{e^{-4(\lambda+\sigma)}}{\lambda + \sigma + \mu}\right]$$

So

$$m_0 = \frac{\frac{\lambda}{\lambda + \sigma}\left[1 - \frac{\mu}{\lambda + \sigma + \mu} e^{-4(\lambda+\sigma)}\right]}{1 + \frac{\lambda\mu}{\lambda + \sigma - \omega}\left[\frac{e^{-4\omega}}{\omega + \mu} - \frac{e^{-4(\lambda+\sigma)}}{\lambda + \sigma + \mu}\right]} \quad \text{S5}$$

Equations S1-S4 show the derived solutions for the proportion of the population seropositive at age 'a'. To make the model more robust to potential predicted trends in seroprevalence especially within the wider age classes in the data, the predicted mean seroprevalence in an age class in the data was calculated explicitly by integrating the predicted seroprevalence across the age class, rather than approximating it by calculating the predicted seroprevalence at the mean age of the age class.

With a constant mortality rate of μ_0 for animals <2 years, and μ ≥2 years, survival at age a is:

$$s(a) = e^{-\mu_0 a} \qquad a < 2 \qquad \text{S6}$$

$$s(a) = e^{-(2\mu_0 + \mu(a-2))} \qquad a \geq 2$$
$$= e^{-\mu a} e^{2(\mu - \mu_0)} \qquad \text{S7}$$

The age distribution is $f(a) = cs(a)$ where c is the normalising constant:

$$c = \frac{1}{\int_0^\infty s(a)\, da}$$

$$\frac{1}{c} = \int_0^\infty s(a)\, da$$
$$= \int_0^2 e^{-\mu_0 a}\, da + e^{2(\mu-\mu_0)} \int_2^\infty e^{-\mu a}\, da$$
$$= \left[-\frac{e^{-\mu_0 a}}{\mu_0}\right]_0^2 + e^{2(\mu-\mu_0)} \left[-\frac{e^{-\mu a}}{\mu}\right]_2^\infty$$
$$= \frac{1 - e^{-2\mu_0}}{\mu_0} + \frac{e^{-2\mu_0}}{\mu}$$
$$= \frac{\mu + (\mu_0 - \mu)e^{-2\mu_0}}{\mu_0 \mu}$$

Meaning that:

$$f(a) = \frac{s(a)\mu_0\mu}{\mu + (\mu_0 - \mu)e^{-2\mu_0}} \qquad \text{S8}$$

The predicted seroprevalence in a representatively sampled age class ranging from a_1 to a_2 is:

$$p(a_1 \to a_2) = \frac{\int_{a1}^{a2} p(a)f(a)\, da}{\int_{a1}^{a2} f(a)\, da} \qquad \text{S9}$$

Taking the predicted seroprevalence at age a for the most complex model (***Equation S4)*** and the age distribution (***Equation S8*** and ***S9****), the predicted seroprevalence in an age class bounded by a1 and a2* becomes:

1. When $a_1 < 2$ and $a_2 < 2$, $f(a) \propto \mu_0 e^{-\mu_0 a}$

$$p(a_1 \rightarrow a_2) = \frac{\left[\frac{\lambda}{\lambda+\sigma}\left(-e^{-\mu_0 a} + \frac{\mu_0 e^{-(\lambda+\sigma+\mu_0)a}}{\lambda+\sigma+\mu_0}\right) - \frac{m_0\,\lambda\mu_0}{\lambda+\sigma-\omega}\left(\frac{e^{-(\omega+\mu_0)a}}{\omega+\mu_0} - \frac{e^{-(\lambda+\sigma+\mu_0)a}}{\lambda+\sigma+\mu_0}\right)\right]_{a_1}^{a_2}}{\left[e^{-\mu_0 a}\right]_{a_1}^{a_2}}$$

$$= \frac{\mu_0}{e^{-\mu_0 a_1} - e^{-\mu_0 a_2}}\left[\frac{\lambda}{\lambda+\sigma}\left(\frac{e^{-\mu_0 a_1} - e^{-\mu_0 a_2}}{\mu_0} - \frac{e^{-(\lambda+\sigma+\mu_0)a_1} - e^{-(\lambda+\sigma+\mu_0)a_2}}{\lambda+\sigma+\mu_0}\right) - \frac{m_0\,\lambda}{\lambda+\sigma-\omega}\left(\frac{e^{-(\omega+\mu_0)a_1} - e^{-(\omega+\mu_0)a_2}}{\omega+\mu_0} - \frac{e^{-(\lambda+\sigma+\mu_0)a_1} - e^{-(\lambda+\sigma+\mu_0)a_2}}{\lambda+\sigma+\mu_0}\right)\right] \qquad \text{S10}$$

2. When $a_1 \geq 2$ and $a_2 \geq 2$, $f(a) \propto \mu e^{-\mu a}$ so $p(a_1 \rightarrow a_2)$ is the same as for ***Equation S10***, substituting μ replacing μ_0

3. When $a_1 < 2$ and $a_2 \geq 2$, the age class is split into two and each band is dealt with separately before averaging the predicted seroprevalence across the whole age class whilst considering the age distribution:

First the age band is split into $a_1 \rightarrow a_2'$ and $a_1' \rightarrow a_2$ where:

$$a_1' = a_2' = 2$$

Then $p(a_1 \rightarrow 2)$ and $p(2 \rightarrow a_2)$ must be calculated, and average appropriately to get $p(a_1 \rightarrow a_2)$ as follows:

$$p(a_1 \rightarrow a_2) = \frac{F_1 p(a_1 \rightarrow 2) + F_2 p(2 \rightarrow a_2)}{F_1 + F_2}$$

where:

$$F_1 = \int_{a_1}^{2} f(a)da = \frac{c}{\mu_0}\left[e^{-\mu_0 a_1} - e^{-2\mu_0}\right]$$

$$F_2 = \int_{2}^{a_2} f(a)da = \frac{ce^{-2\mu_0}}{\mu}\left[1 - e^{-\mu(a_2-2)}\right]$$

so:

$$p(a_1 \rightarrow a_2) = \frac{\mu[e^{-\mu_0 a_1} - e^{-2\mu_0}]p(a_1 \rightarrow 2) + \mu_0 e^{-2\mu_0}[1 - e^{-\mu(a_2-2)}]p(2 \rightarrow a_2)}{\mu[e^{-\mu_0 a_1} - e^{-2\mu_0}] + \mu_0 e^{-2\mu_0}[1 - e^{-\mu(a_2-2)}]} \quad \text{S11}$$

Similarly, the proportion of animals with maternally acquired antibodies, when constant mortality rates are considered, is as follows:

When $a_1 < 2, a_2 < 2$:

$$m(a_1 \rightarrow a_2) = \frac{\mu_0 m_0 \left(e^{-(\omega+\mu_0)a_1} - e^{-(\omega+\mu_0)a_2}\right)}{(\omega + \mu_0)(e^{-\mu_0 a_1} - e^{-\mu_0 a_2})} \quad \text{S12}$$

When $a_1 \geq 2, a_2 \geq 2$:

$$m(a_1 \rightarrow a_2) = \frac{\mu m_0 \left(e^{-(\omega+\mu)a_1} - e^{-(\omega+\mu)a_2}\right)}{(\omega + \mu)(e^{-\mu a_1} - e^{-\mu a_2})} \quad \text{S13}$$

And when $a_1 < 2, a_2 \geq 2$:

$$m(a_1 \rightarrow a_2) = \frac{F_1 m(a_1 \rightarrow 2) + F_2 m(2 \rightarrow a_2)}{F_1 + F_2} \quad \text{S14}$$

4. Catalytic modelling - reparameterisation of the beta-binomial distribution

The MERS-CoV seroprevalence studies were conducted across a range of camel populations in different locations and herd management systems and used different assays to determine seropositivity. To account for this potential overdispersion we assumed that the seroprevalence data was beta-binomially distributed, so that the number of seropositive animals in age class i X_i follows a beta-binomial distribution defined as:

$$X_i \sim BetaBinomial(N_i, p_i, k) \quad \text{S15}$$

where k represents the amount of overdispersion in the data.

The likelihood of the beta-binomial model can be defined by the probability mass function of the beta-binomial distribution:

$$L(p_i) = \binom{N_i}{X_i} \frac{B(X_i + \alpha,\ N_i - X_i + \beta)}{B(\alpha, \beta)} \quad \text{S16}$$

where α and β are the standard parameters of the beta distribution and are both >0:

However, for our purposes it was useful to re-parameterise the beta-binomial distribution in terms of the mean probability of being seropositive, p, and an overdispersion parameter, k.

$$p = \frac{\alpha}{\alpha + \beta} \quad \text{S17}$$

$$k = \frac{n - 1}{\alpha + \beta + 1} \quad \text{S18}$$

The parameter $k > 0$, and a k approaching zero would indicate negligible overdispersion. With this reparameterisation, the variance of the beta-binomial distribution becomes:

$$var[X] = np(1 - p)\{1 + k\} \quad \text{S19}$$

Note that the first term in ***S19***, $np(1 - p)$, is equal to the variance of binomially distributed observations meaning the reparameterization results in a quasi-binomial parameterisation for the beta binomial, in which binomial variance is scaled by a fixed constant $1 + k$. This gives a weaker overdispersion than the more standard reparameterisation of the beta-binomial in which overdispersion scales with n^2 and so offers better control of overdispersion through the use of priors. A normal distribution with a mean of zero and a standard deviation of 0.5, restricted to positive values (a "half-normal" distribution), was used as the prior for the overdispersion parameter. Priors for k with greater standard deviations up to 2 were also used to test whether the results were robust to the choice of prior.

5. Catalytic model selection

Once maternally acquired antibodies (mAbs) were included in the catalytic model, allowing for seroreversion in Model 4 afforded a small additional improvement in model file. The relative underestimation of the effective number of parameters in model 3 compared to model 4 (***Table S1, S2***) likely means that model 3 is under-penalised by DIC which may contribute to minimizing the relative amount of improvement afforded by seroreversion in model 4.

Table S1. *Model comparison using Deviance Information Criterion (DIC) under our central assumptions (sensitivity = 98.0% for neutralisation tests (NTs), 98.0% for non-neutralisation tests. specificity = 99.5% for NTs, 98.5% for non-NTs).*

	DIC	Effective number of parameters, pD
model 1: seroconversion	533	19
model 2: seroconversion + seroreversion	507	20
model 3: seroconversion + mAbs	493	15
model 4: seroconversion + seroreversion + mAbs	492	21

Table S2. *Model comparison using Deviance Information Criterion (DIC) with alternative test sensitivity and specificity (sensitivity = 98.0% for neutralisation tests (NTs), 98.0% for non-neutralisation tests. specificity = 85.0% for NTs, 98.5% for non-NTs).*

	DIC	Effective number of parameters, pD
model 1: seroconversion	508	17
model 2: seroconversion + seroreversion	502	17
model 3: seroconversion + mAbs	499	16
model 4: seroconversion + seroreversion + mAbs	497	18

6. Leave One Out (LOO) analysis of catalytic model selection and parameter estimation

To check the influence of each real data set on the global parameters, each data set was removed in turn and the parameters were re-estimated. The best fitting model was Model 4 or Model 3 regardless of the exclusion of any dataset (***Figure S2***). The parameter estimates were relatively consistent when each of the 23 datasets were removed with two notable exceptions. Firstly, for model 4 omitting any of the datasets measuring seroprevalence in Kenya slightly increased estimates for the rate of waning of mAbs, ω, and Abs, σ (***Figure S3***). Secondly, the removal of the seroprevalence in dromedaries in Egypt[6] decreased the rate of waning of mAbs so that it was similar to that in model 3, and decreased the estimated overdispersion parameter. The study in question measured a much lower seroprevalence measure for young calves compared to the other studies included in model fitting. This difference likely explains the dataset's effect on ω and on the overdispersion parameter. When we ran a sensitivity analysis which assumed an alternative value for ω based on the Model 4 estimate when the Egypt study was excluded, R_0 estimates were similar for most populations (difference of <1.5) with the exception of those with very high FoI as a result of limitations due to seroprevalence approaching 100, in which R_0 values increased.

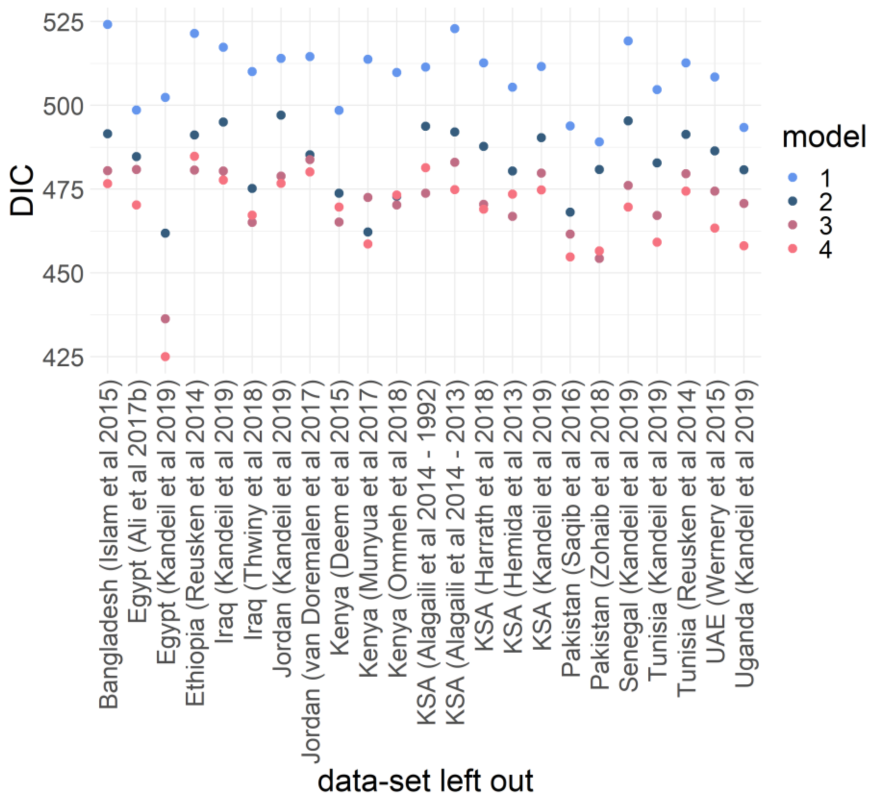

Figure S2. *Comparison of the Deviance Information Criterion (DIC) across models when each dataset is left out in turn (model 1: seroconversion, model 2: seroconversion + seroreversion, model 3: seroconversion + mAbs, model 4: seroconversion + mAbs + seroreversion). The best fitting model is model 3 or 4 in all cases.*

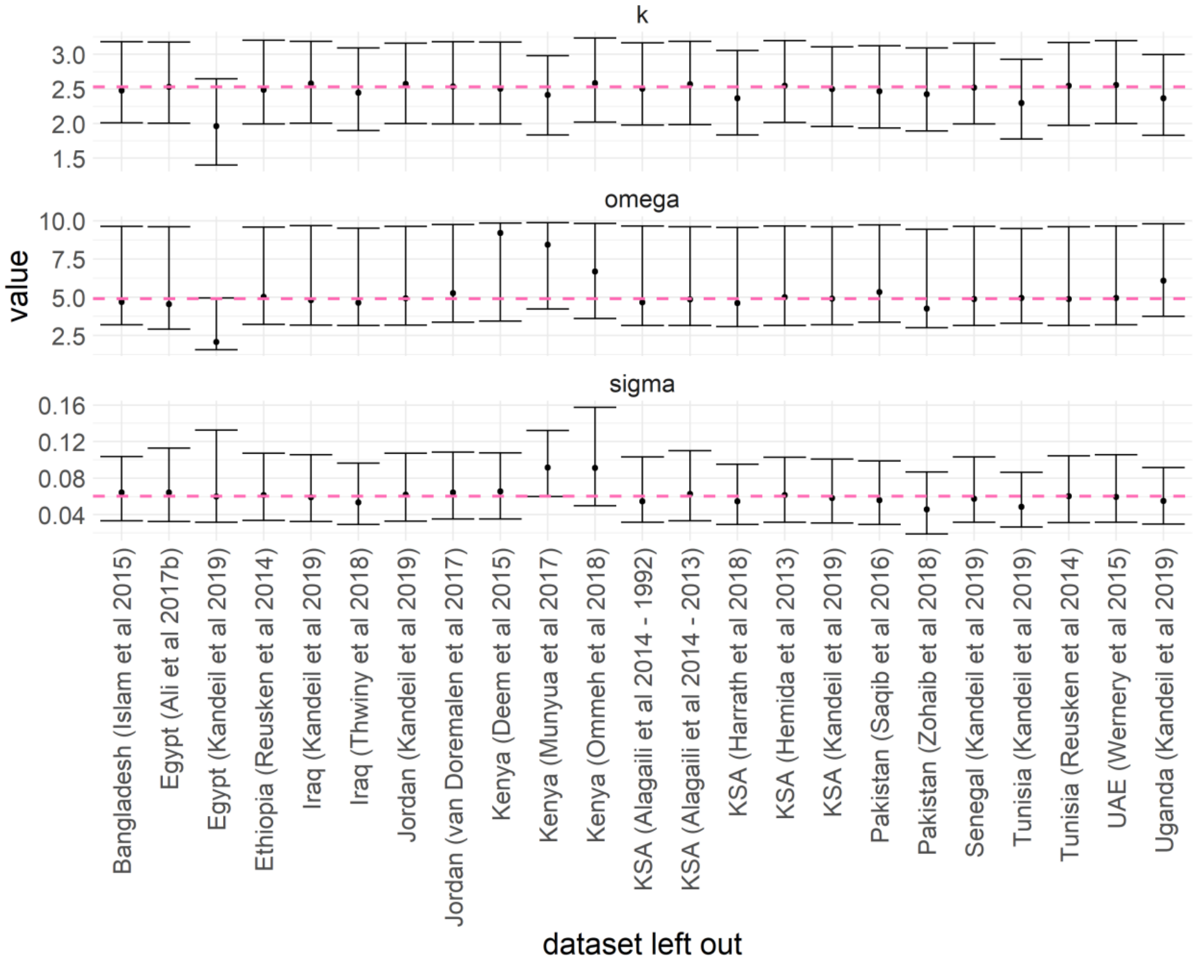

Figure S3. *Comparison of the model 4 estimates of the overdispersion parameter, k, the rate of waning of mAbs, ω, and the rate of waning of Abs following infection, σ, when each dataset is left out in turn. Error bars represent 95% CrIs based on n = 10,000 posterior samples. The pink dotted line represents the central estimates when all datasets are included.*

***Table S3**. FoI estimates for all 4 sero-catalytic models, and for two alternative test sensitivity assumptions. Estimates presented are the mode and 95% CrI.*

		Sensitivity = **98.0% NT**, 98.0% Non-NT. Specificity = 99.5% NT, 98.5% Non-NT				Sensitivity = **85.0% NT**, 98.0% Non-NT. Specificity = 99.5% NT, 98.5% Non-NT			
	FoI, λ	Model 1	Model 2	Model 3	Model 4	Model 1	Model 2	Model 3	Model 4
Africa	Egypt [5]	0.36 (0.31, 0.42)	0.65 (0.52, 0.86)	0.35 (0.3, 0.4)	0.51 (0.42, 0.68)	0.74 (0.59, 1.02)	0.85 (0.68, 1.16)	0.68 (0.55, 0.93)	0.79 (0.62, 1.07)
	Egypt [6]	0.33 (0.28, 0.39)	0.57 (0.46, 0.74)	0.33 (0.28, 0.39)	0.47 (0.38, 0.61)	0.53 (0.43, 0.67)	0.61 (0.5, 0.8)	0.52 (0.43, 0.67)	0.59 (0.48, 0.78)
	Ethiopia [9]	1.61 (1, 9.68)	2.87 (1.68, 9.72)	2.41 (1.34, 9.69)	2.66 (1.56, 9.69)	1.66 (1.02, 9.68)	1.89 (1.19, 9.66)	1.96 (1.15, 9.67)	2.06 (1.27, 9.66)
	Kenya [10]	0.17 (0.11, 0.28)	0.29 (0.19, 0.49)	0.16 (0.11, 0.25)	0.24 (0.16, 0.39)	0.17 (0.11, 0.27)	0.2 (0.13, 0.34)	0.17 (0.12, 0.27)	0.19 (0.13, 0.32)
	Kenya [11]	0.62 (0.49, 0.87)	1.28 (0.92, 7.7)	0.73 (0.57, 1.03)	1.01 (0.76, 2.33)	0.63 (0.5, 0.87)	0.76 (0.59, 1.2)	0.67 (0.53, 0.94)	0.78 (0.6, 1.18)
	Kenya [12]	0.18 (0.15, 0.21)	0.38 (0.29, 0.56)	0.17 (0.15, 0.21)	0.29 (0.22, 0.43)	0.17 (0.15, 0.21)	0.23 (0.18, 0.32)	0.18 (0.15, 0.21)	0.22 (0.17, 0.3)
	Senegal [6]	0.2 (0.13, 0.35)	0.35 (0.23, 0.77)	0.2 (0.14, 0.35)	0.3 (0.19, 0.58)	0.31 (0.2, 7.7)	0.38 (0.24, 7.16)	0.3 (0.2, 6.98)	0.36 (0.23, 6.6)
	Tunisia [6]	0.46 (0.36, 3.38)	1.18 (0.78, 3.85)	0.49 (0.39, 0.72)	0.82 (0.59, 2.21)	9.65 (2.96, 9.89)	9.67 (3.21, 9.9)	9.63 (2.89, 9.89)	9.66 (3.08, 9.89)
	Tunisia [9]	0.13 (0.08, 0.22)	0.21 (0.13, 0.37)	0.13 (0.08, 0.22)	0.18 (0.11, 0.31)	0.13 (0.08, 0.21)	0.15 (0.09, 0.26)	0.13 (0.08, 0.22)	0.15 (0.09, 0.26)
	Uganda [6]	0.24 (0.17, 0.36)	0.46 (0.31, 0.77)	0.22 (0.17, 0.33)	0.34 (0.24, 0.56)	0.63 (0.37, 3.49)	0.74 (0.43, 2.68)	0.42 (0.28, 2.06)	0.52 (0.34, 1.96)
Middle East	Iraq [6]	0.21 (0.08, 8.64)	0.33 (0.14, 7.99)	0.16 (0.07, 7.58)	0.24 (0.1, 7.49)	0.45 (0.21, 9.64)	0.49 (0.23, 9.63)	0.4 (0.18, 9.64)	0.43 (0.19, 9.61)
	Iraq [13]	5.95 (1.4, 9.77)	2.48 (1.2, 9.42)	3.55 (0.73, 9.71)	1.77 (0.92, 9.43)	5.59 (1.44, 9.77)	3.95 (1.12, 9.67)	5.84 (0.96, 9.75)	3.08 (0.89, 9.65)
	Jordan [6]	0.71 (0.48, 2.13)	1.27 (0.83, 3.12)	0.66 (0.45, 1.54)	1.03 (0.67, 2.43)	1.5 (0.9, 8.02)	1.69 (1.04, 8.6)	1.41 (0.87, 7.91)	1.59 (0.97, 8.48)
	Jordan [14]	1.96 (0.96, 8.83)	2.46 (1.17, 9.18)	2.1 (0.99, 9.36)	2.63 (1.2, 9.46)	2.05 (0.98, 8.85)	2.13 (1.04, 8.98)	2.33 (1.07, 9.37)	2.44 (1.12, 9.41)
	KSA [15] 1992-2010	2.1 (1, 8.9)	2.28 (1.37, 8.35)	1.18 (0.72, 8.04)	1.84 (1.09, 7.75)	2.13 (1.01, 8.81)	1.95 (1.09, 8.4)	1.58 (0.83, 8.53)	1.75 (0.95, 8.16)
	KSA [15] 2013	1.02 (0.61, 2.86)	1.41 (0.88, 3.54)	0.82 (0.5, 2.18)	1.15 (0.71, 2.84)	1.03 (0.62, 2.79)	1.13 (0.7, 2.9)	0.9 (0.54, 2.46)	1.01 (0.62, 2.63)
	KSA [16]	4.8 (1.52, 9.75)	2.51 (1.31, 9.52)	3.51 (1, 9.67)	2.29 (1.15, 9.47)	4.88 (1.54, 9.77)	2.94 (1.32, 9.68)	3.77 (1.24, 9.71)	2.86 (1.17, 9.63)
	KSA [17]	3.72 (1.86, 9.54)	3.76 (2.16, 9.38)	2.48 (1.45, 9.16)	3.03 (1.74, 9.12)	7.63 (2.85, 9.83)	8.55 (3.1, 9.84)	7.35 (2.67, 9.82)	8.56 (2.89, 9.84)
	KSA [6]	0.34 (0.23, 1.39)	0.71 (0.45, 3.61)	0.36 (0.24, 0.76)	0.55 (0.36, 2.17)	5.23 (0.9, 9.77)	4.49 (1, 9.76)	4.62 (0.85, 9.76)	4.38 (0.94, 9.76)
	UAE [18]	8.2 (3.89, 9.86)	8.1 (4.1, 9.86)	7.07 (2.86, 9.81)	7.1 (3.48, 9.82)	8.52 (3.94, 9.87)	7.84 (3.79, 9.85)	7.89 (3.52, 9.85)	7.42 (3.44, 9.83)
South Asia	Bangladesh [19]	0.08 (0.03, 0.26)	0.13 (0.05, 0.48)	0.07 (0.03, 0.22)	0.1 (0.04, 0.34)	0.1 (0.04, 2.34)	0.12 (0.05, 1.95)	0.09 (0.04, 1.02)	0.11 (0.05, 1.13)
	Pakistan [20]	0.1 (0.07, 0.13)	0.16 (0.12, 0.24)	0.1 (0.07, 0.13)	0.13 (0.1, 0.19)	0.13 (0.09, 0.18)	0.15 (0.11, 0.22)	0.13 (0.09, 0.18)	0.15 (0.1, 0.21)
	Pakistan [21]	0.3 (0.25, 0.38)	0.56 (0.42, 0.84)	0.31 (0.26, 0.39)	0.45 (0.35, 0.65)	0.3 (0.25, 0.38)	0.37 (0.3, 0.5)	0.3 (0.25, 0.38)	0.36 (0.29, 0.48)
Global	Rate of waning mAbs, ω	NA	NA	2.82 (2.14, 4.79)	4.9 (3.19, 9.64)	NA	NA	5.3 (3.37, 9.7)	6.82 (3.88, 9.84)
	Rate of waning Abs, σ	NA	0.1 (0.07, 0.16)	NA	0.06 (0.03, 0.11)	NA	0.03 (0.01, 0.05)	NA	0.02 (0.01, 0.05)
	Overdispersion, k	2.92 (2.42, 3.54)	2.79 (2.23, 3.41)	2.52 (2.01, 3.14)	2.53 (1.98, 3.17)	2.8 (2.27, 3.4)	2.76 (2.24, 3.38)	2.7 (2.16, 3.3)	2.67 (2.15, 3.3)

Table S4. *R_0 estimates under different immunity assumptions (top 4 rows). Estimates presented propagate the uncertainty in the FoI using the 95% CrIs based on n = 10,000 posterior samples.*

**Parameter set***	$1/\sigma$	30 days	30 days	30 days	30 days	30 days	30 days	90 days	90 days	1 day	1 day	30 days	30 days
	φ	75%	75%	100%	100%	25%	25%	75%	75%	75%	75%	75%	75%
	r_{inf}	1%	50%	1%	50%	1%	50%	1%	50%	1%	50%	1%	50%
	$1/\omega$	2.4 months	2.4 months	2.4 months	2.4 months	2.4 months	2.4 months	2.4 months	2.4 months	2.4 months	2.4 months	4.2 months	4.2 months
Africa	Egypt[5]	4.2 (3.7, 5.1)	1.9 (1.8, 2.1)	4.2 (3.6, 5.1)	1.7 (1.6, 1.8)	4.3 (3.7, 5.3)	3.1 (2.8, 3.5)	4.2 (3.7, 5.2)	2 (1.8, 2.3)	4.2 (3.7, 5.1)	1.9 (1.8, 2)	3.4 (3.1, 3.7)	1.8 (1.8, 1.9)
	Egypt[6]	4 (3.5, 4.7)	1.9 (1.8, 2)	3.9 (3.4, 4.7)	1.7 (1.6, 1.7)	4 (3.5, 4.8)	2.9 (2.6, 3.3)	4 (3.5, 4.8)	1.9 (1.8, 2.2)	4 (3.5, 4.7)	1.8 (1.8, 1.9)	3.3 (3, 3.6)	1.8 (1.7, 1.9)
	Ethiopia[9]	14.9 (9.6, 44)	3 (2.6, 4.9)	14.6 (9.4, 42.9)	2.5 (2.1, 4.3)	15.7 (9.9, 48.3)	5.9 (4.9, 9.1)	15.2 (9.6, 47.1)	3.6 (2.9, 7.6)	14.7 (9.5, 40.8)	2.6 (2.4, 3.5)	14.3 (8.9, 45.8)	3 (2.5, 5)
	Kenya[10]	2.6 (2.1, 3.5)	1.7 (1.4, 1.8)	2.6 (2.1, 3.5)	1.4 (1.3, 1.6)	2.6 (2.1, 3.6)	2.2 (1.8, 2.7)	2.6 (2.1, 3.5)	1.5 (1.3, 1.8)	2.6 (2.1, 3.5)	1.6 (1.4, 1.8)	2.2 (2.2, 2.8)	1.5 (1.4, 1.7)
	Kenya[11]	6.8 (5.5, 13.4)	2.3 (2.2, 2.9)	6.8 (5.5, 13.1)	1.9 (1.8, 2.4)	7 (5.7, 13.9)	4.1 (3.6, 5.7)	6.9 (5.6, 13.5)	2.5 (2.3, 3.4)	6.8 (5.5, 13.1)	2.2 (2.1, 2.5)	5.6 (4.7, 7.3)	2.2 (2.1, 2.4)
	Kenya[12]	2.9 (2.5, 3.7)	1.7 (1.6, 1.9)	2.9 (2.5, 3.7)	1.5 (1.4, 1.6)	3 (2.5, 3.8)	2.4 (2.1, 2.8)	2.9 (2.5, 3.7)	1.6 (1.5, 1.8)	2.9 (2.5, 3.7)	1.7 (1.6, 1.8)	2.2 (2.2, 2.5)	1.5 (1.5, 1.6)
	Senegal[6]	2.9 (2.3, 4.6)	1.7 (1.5, 2)	2.9 (2.3, 4.6)	1.5 (1.3, 1.7)	3 (2.3, 4.7)	2.4 (2, 3.3)	2.9 (2.3, 4.6)	1.6 (1.4, 2.1)	2.9 (2.3, 4.6)	1.7 (1.5, 1.9)	2.4 (2.2, 3.4)	1.6 (1.4, 1.8)
	Tunisia[6]	5.8 (4.6, 12.8)	2.2 (2, 2.8)	5.8 (4.6, 12.6)	1.8 (1.7, 2.3)	6 (4.7, 13.3)	3.7 (3.3, 5.6)	5.9 (4.6, 12.9)	2.4 (2.1, 3.4)	5.8 (4.6, 12.6)	2.1 (1.9, 2.5)	4.3 (3.6, 5.6)	2 (1.9, 2.2)
	Tunisia[9]	2.2 (1.8, 3.1)	1.5 (1.3, 1.7)	2.2 (1.7, 3)	1.3 (1.2, 1.5)	2.2 (1.8, 3.1)	1.9 (1.6, 2.4)	2.2 (1.8, 3.1)	1.4 (1.3, 1.6)	2.2 (1.8, 3.1)	1.5 (1.3, 1.7)	2.2 (2.2, 2.6)	1.4 (1.3, 1.7)
	Uganda[6]	3.2 (2.6, 4.5)	1.8 (1.7, 2)	3.2 (2.6, 4.4)	1.5 (1.4, 1.7)	3.2 (2.6, 4.6)	2.5 (2.2, 3.2)	3.2 (2.6, 4.5)	1.7 (1.5, 2.1)	3.2 (2.6, 4.5)	1.7 (1.6, 1.9)	2.6 (2.2, 3.3)	1.7 (1.5, 1.8)
Middle East	Iraq[6]	2.6 (1.7, 35.3)	1.7 (1.3, 4.3)	2.6 (1.7, 34.3)	1.4 (1.2, 3.7)	2.6 (1.7, 38.4)	2.2 (1.5, 8.4)	2.6 (1.7, 37.2)	1.5 (1.3, 6.4)	2.6 (1.7, 33.3)	1.6 (1.3, 3.2)	2.2 (2.2, 37.1)	1.5 (1.2, 4.4)
	Iraq[13]	10.6 (6.4, 43)	2.7 (2.3, 4.8)	10.4 (6.3, 41.9)	2.2 (1.9, 4.2)	11 (6.6, 47.1)	5.1 (3.9, 9)	10.7 (6.4, 46)	3.1 (2.4, 7.4)	10.5 (6.4, 39.9)	2.4 (2.2, 3.4)	19.6 (5.7, 45.9)	3.3 (2.2, 5)
	Jordan[6]	6.9 (5, 13.8)	2.3 (2.1, 2.9)	6.8 (5, 13.6)	1.9 (1.8, 2.4)	7.1 (5.2, 14.5)	4.1 (3.5, 5.8)	6.9 (5.1, 14)	2.5 (2.3, 3.5)	6.9 (5, 13.6)	2.2 (2, 2.5)	5.2 (4, 9.9)	2.2 (1.9, 2.6)
	Jordan[14]	14.8 (7.8, 43.1)	3 (2.4, 4.8)	14.5 (7.7, 41.9)	2.5 (2, 4.2)	15.5 (8, 47.2)	5.9 (4.4, 9)	15 (7.8, 46.1)	3.6 (2.6, 7.4)	14.5 (7.7, 40)	2.6 (2.3, 3.4)	12.8 (7, 44.4)	2.9 (2.4, 4.9)
	KSA[15] 1992-2010	10.9 (7.2, 36.3)	2.7 (2.4, 4.4)	10.8 (7.1, 35.3)	2.2 (1.9, 3.8)	11.3 (7.4, 39.6)	5.2 (4.2, 8.5)	11 (7.3, 38.4)	3.1 (2.6, 6.5)	10.8 (7.2, 34.2)	2.4 (2.3, 3.2)	8 (5.6, 39)	2.5 (2.2, 4.6)
	KSA[15] 2013	7.5 (5.3, 15.7)	2.4 (2.1, 3)	7.4 (5.2, 15.4)	2 (1.8, 2.5)	7.7 (5.4, 16.6)	4.3 (3.5, 6.1)	7.5 (5.3, 16)	2.6 (2.3, 3.7)	7.5 (5.3, 15.5)	2.3 (2, 2.6)	6.1 (4.3, 13.2)	2.3 (2, 2.9)
	KSA[16]	13.2 (7.5, 43.1)	2.9 (2.4, 4.8)	12.9 (7.4, 42)	2.4 (2, 4.2)	13.7 (7.8, 47.3)	5.7 (4.4, 9.1)	13.3 (7.6, 46.1)	3.4 (2.6, 7.4)	12.9 (7.5, 40)	2.5 (2.3, 3.4)	19.4 (7.1, 45.7)	3.3 (2.4, 5)
	KSA[17]	16.6 (10.4, 41.7)	3.1 (2.6, 4.7)	16.2 (10.3, 40.6)	2.6 (2.2, 4.1)	17.5 (10.8, 45.7)	6.2 (5.1, 8.9)	16.9 (10.6, 44.6)	3.9 (3, 7.2)	16.3 (10.3, 38.9)	2.7 (2.4, 3.4)	14.6 (9.4, 43.6)	3 (2.6, 4.9)
	KSA[6]	4.5 (3.4, 12.6)	2 (1.8, 2.8)	4.4 (3.3, 12.4)	1.7 (1.6, 2.3)	4.5 (3.4, 13.1)	3.2 (2.6, 5.6)	4.5 (3.4, 12.7)	2.1 (1.7, 3.3)	4.4 (3.4, 12.4)	1.9 (1.8, 2.5)	3.4 (2.7, 5.8)	1.8 (1.7, 2.3)
	UAE[18]	33.7 (18.5, 44.5)	4.2 (3.2, 4.9)	32.8 (18.1, 43.4)	3.6 (2.7, 4.3)	36.6 (19.7, 48.8)	8.2 (6.5, 9.2)	35.5 (19, 47.7)	6.1 (4.1, 7.6)	31.9 (18.1, 41.2)	3.2 (2.7, 3.5)	34.9 (16.4, 46.3)	4.3 (3.1, 5)
South Asia	Bangladesh[19]	1.7 (1.3, 3.2)	1.3 (1.1, 1.8)	1.7 (1.3, 3.2)	1.2 (1.1, 1.5)	1.7 (1.3, 3.3)	1.5 (1.2, 2.5)	1.7 (1.3, 3.2)	1.2 (1.1, 1.7)	1.7 (1.3, 3.2)	1.3 (1.1, 1.7)	2.2 (2.2, 2.5)	1.2 (1.1, 1.7)
	Pakistan[20]	1.9 (1.7, 2.3)	1.4 (1.3, 1.5)	1.9 (1.7, 2.3)	1.2 (1.2, 1.3)	1.9 (1.7, 2.3)	1.7 (1.5, 2)	1.9 (1.7, 2.3)	1.3 (1.2, 1.4)	1.9 (1.7, 2.3)	1.4 (1.3, 1.5)	2.2 (2.2, 2.2)	1.3 (1.2, 1.4)
	Pakistan[21]	3.9 (3.3, 4.9)	1.9 (1.8, 2.1)	3.8 (3.3, 4.9)	1.7 (1.5, 1.8)	3.9 (3.3, 5)	2.9 (2.6, 3.4)	3.9 (3.3, 5)	1.9 (1.7, 2.2)	3.9 (3.3, 4.9)	1.8 (1.7, 2)	3.1 (2.8, 3.6)	1.8 (1.7, 1.9)

*$1/\sigma$ = *duration of complete immunity following infection.* Φ = *relative susceptibility following complete immunity.* r_{inf} = *relative infectiousness of reinfected animals.* $1/\omega$ = *duration of mAbs.*

Transmission modelling and simulating vaccine impact

1. Selection of R_0 values to take forward into dynamic modelling simulations

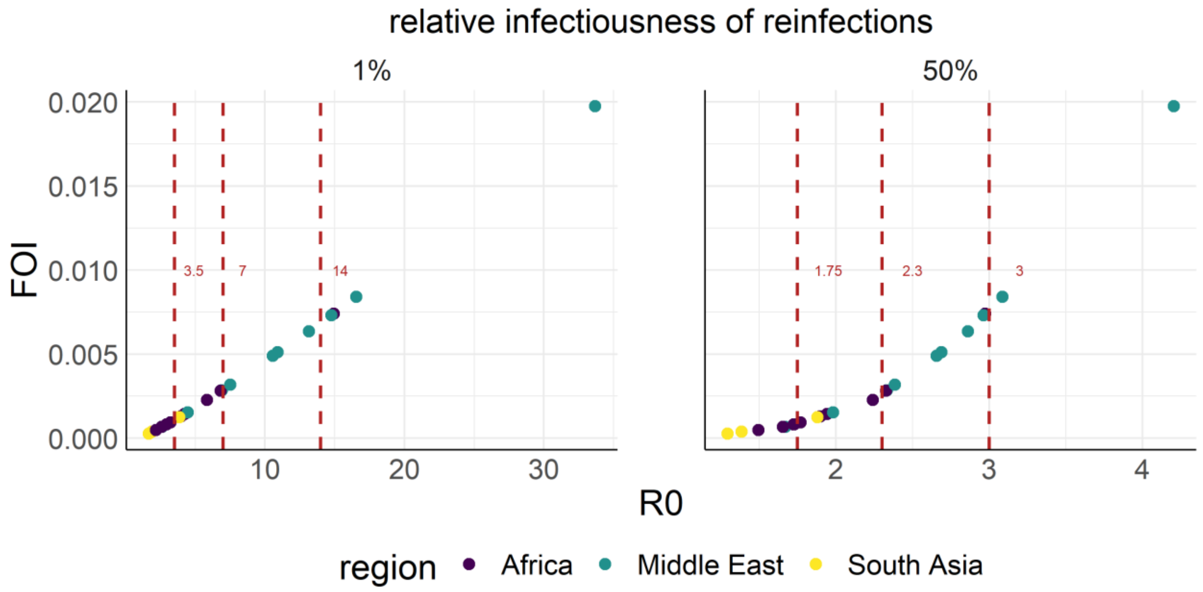

Figure S4 *The three R_0 values chosen to represent the full span of inferred transmissibility across the 23 studies of age-stratified seroprevalence shown as red dashed lines. Assumptions about the relative infectiousness of reinfected individuals changes how the Force of Infection (FoI) translates to R_0 so two options are shown – the selection for our central analysis assuming viral load is proportional to infectiousness so reinfections as 1% as infectious as first-time infections (left), and secondly our sensitivity analysis where we assume viral load is proportional to the logarithm of infectiousness and so reinfections are 50% as infectious as first-time infections (right).*

2. Metapopulation structure and modelling external contributions of FoI

For large populations, it becomes unrealistic to assume populations are well mixed, but the movements and interactions between herds of dromedaries are not well documented. In order to explore the effect population structure has on dynamics, we developed a rudimentary structured population model where sub-populations or patches are arranged over a grid (***Figure S5***). Individuals are most likely to be in contact with other individuals in the same patch, less likely to meet individuals in neighbouring patches, and do not meet individuals in distant patches. Patches experience different degrees of connection depending on whether they are in a corner, on edge or within the middle of the grid. In reality, herds will also have varying levels of connection to other herds or larger sub-populations.

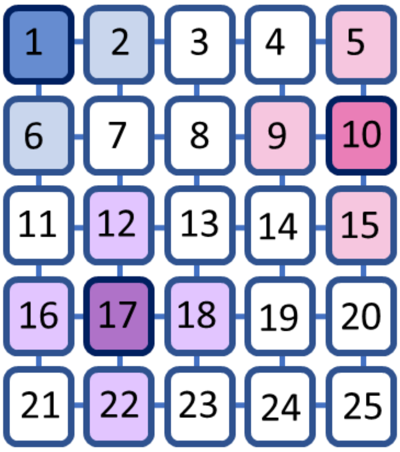

Figure S5. *A structured population. A grid of 5x5 patches is used to represent herds or larger sub-populations connected to their nearest neighbours. Individuals in herds in the corners such as in herd 1 (in dark blue) have contact with individuals in 2 neighbouring herds (light blue). Individuals in herds on grid edges such as herd 10 (dark pink) have contact with individuals in 3 neighbouring herds (light pink), and individuals in internal herds such as herd 17 (dark purple) have contact with individuals in 4 neighbouring herds (light purple).*

The connections between neighbouring patches are modelled as external contributions to the FoI experienced by susceptible individuals in each patch. For example, if for simplicity we ignore reinfections and assume a single infectious state I, then the FoI, l^i, experienced by a completely susceptible individual in herd *i* in ***Figure S5*** is as follows:

For herd 1 – a corner connected to two neighbouring patches:

$$\lambda^1 = (1-2\upsilon)\frac{\beta I^1}{N^1} + \upsilon\left(\frac{\beta I^2}{N^2} + \frac{\beta I^6}{N^6}\right) \quad \text{S20}$$

For herd 10 – on an edge with three neighbouring patches:

$$\lambda^{10} = (1-3\upsilon)\frac{\beta I^{10}}{N^{10}} + \upsilon\left(\frac{\beta I^5}{N^5} + \frac{\beta I^9}{N^9} + \frac{\beta I^{15}}{N^{15}}\right) \quad \text{S21}$$

For herd 17 – within the middle of the grid with four neighbouring patches:

$$\lambda^{17} = (1-4\upsilon)\frac{\beta I^{17}}{N^{17}} + \upsilon\left(\frac{\beta I^{12}}{N^{12}} + \frac{\beta I^{16}}{N^{16}} + \frac{\beta I^{18}}{N^{18}} + \frac{\beta I^{22}}{N^{22}}\right) \quad \text{S22}$$

Where β is the effective contact rate, I^i is the number of infectious individuals in herd *i*, N^i is the total number of individuals in herd *i*, and υ represents the strength of connection between neighbouring herds. If υ = 0.01, this means that 1% of a dromedary's contacts are from each of their neighbouring herds and the remainder of their contacts are within their own herd. So, for example a total of 2% of the contacts of a dromedary in herd 1 are with dromedaries from neighbouring herds and 98% are with other dromedaries within herd 1.

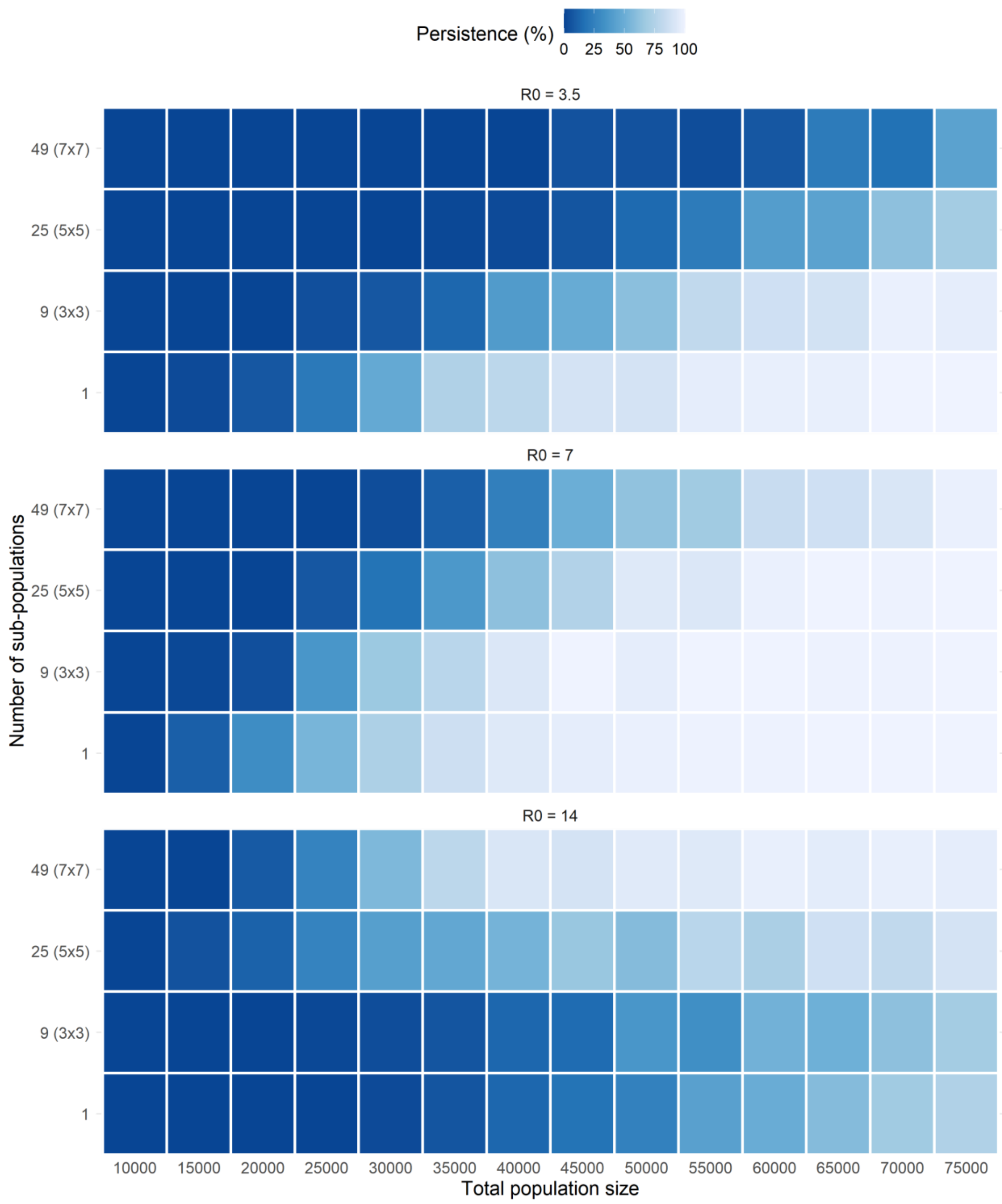

Figure S6. *The effect of the degree of population structure on the percentage of stochastic model runs in which transmission persists for at least 25 years. Persistence is shown for a given total population size (x axis) broken down into increasing number of smaller sub-populations (y axis), for three different transmission intensities. At low (R_0 = 3.5) and moderate (R_0 = 7) transmission intensities the increasing structure in the population reduces persistence, whereas at a high transmission intensity (R_0 = 14) the increasing structure increases persistence. This analysis was run assuming reinfections are 1% as infections as reinfections and births reflect the full degree of seasonality representative of calving in Kingdom of Saudi Arabia.*

3. Vaccine impact

a. Incidence in older animals following vaccination of young calves

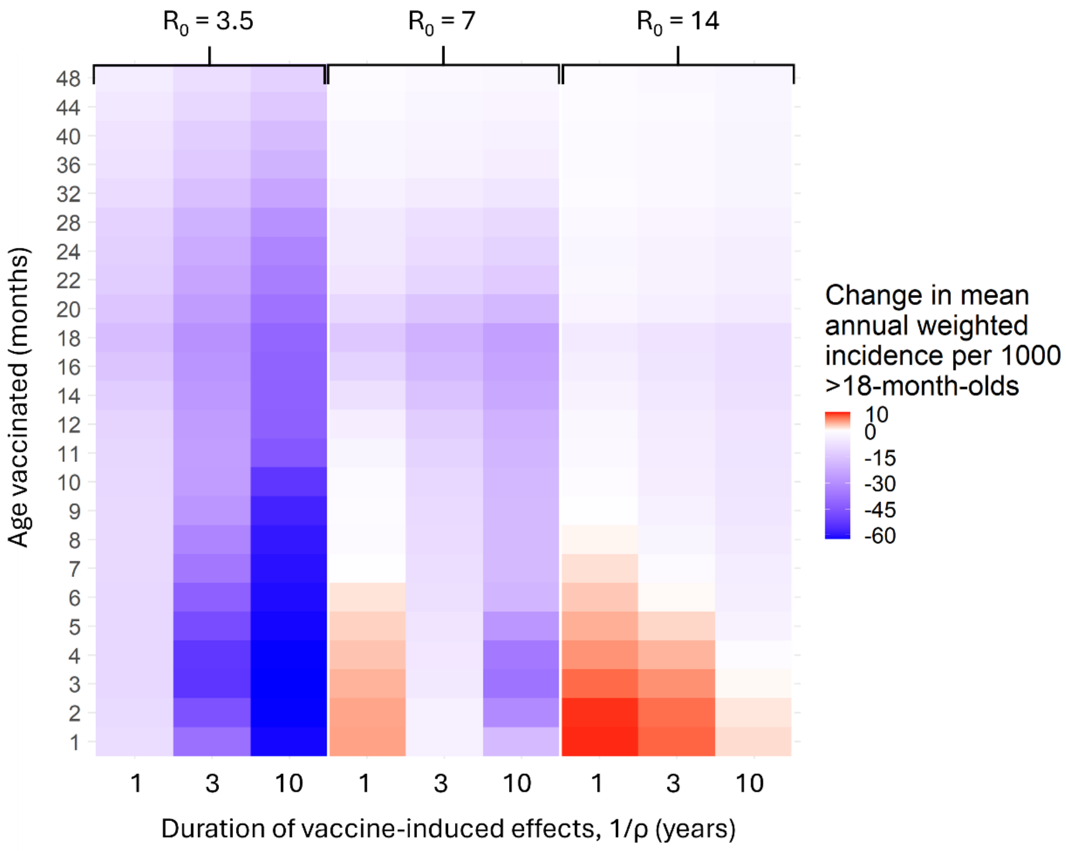

Figure S7. *The average change in annual infectious-state-weighted incidence in dromedaries older than 18 months per 1,000 animals in the ten years following vaccine introduction compared to in the absence of vaccination. Infectious-state-weighted incidence considers that vaccinated and previously infected animals are less infectious than first-time infected animals and weights incidence in those animals proportional to their infectiousness to give a measure of incidence that is more relevant when considering spillover risk. Red denotes increases and blue denotes decreases in total incidence in animals >18-months old.*

b. Optimal age for reducing overall incidence – sensitivity analysis under different efficacy scenarios

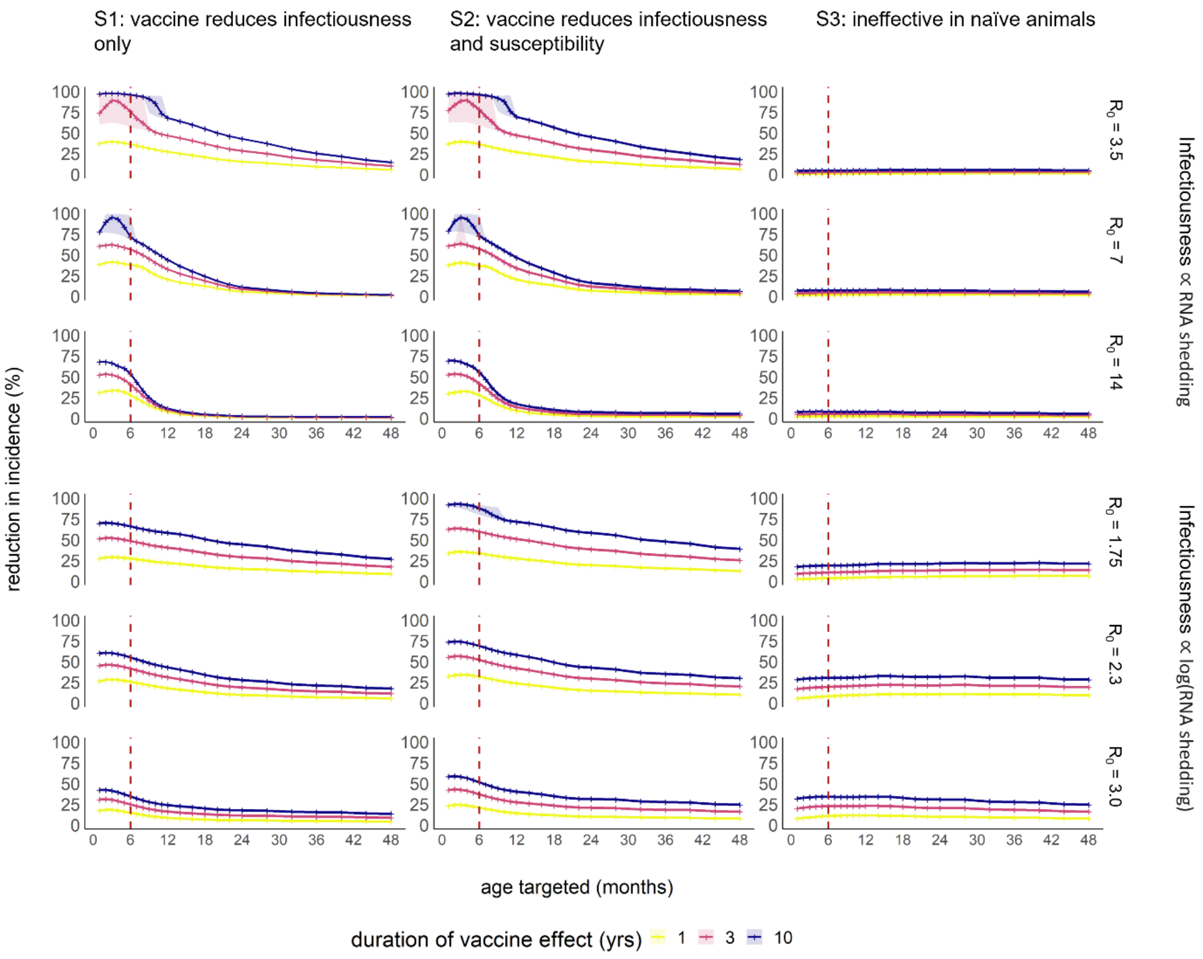

Figure S8. *The percentage reduction in incidence of MERS-CoV infection in camels depending on age group targeted, in results for different transmission intensities spanning estimates from age-stratified seroprevalence data, and under different vaccine efficacy scenarios. Transparent ribbons show the 2.5-97.5% quantiles across n = 1000 stochastic model runs, with stochastic fadeout of transmission leading to larger differences across stochastic runs in some scenarios. The red dashed line indicates the 6-month age class. Vaccine coverage was assumed to be 80% in the target age class.*

c. *Impact of vaccination by coverage under different efficacy scenarios*

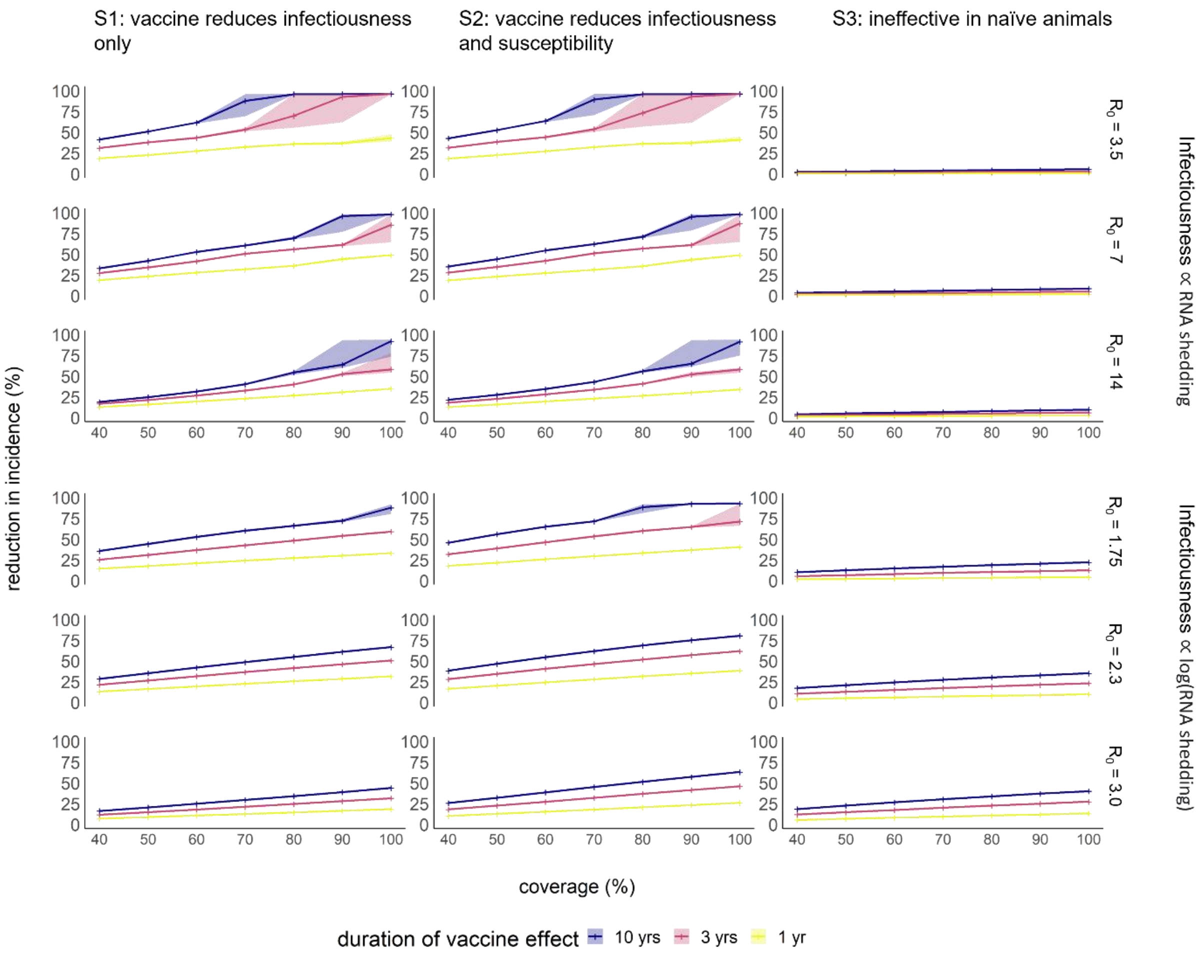

Figure S9. *The percentage reduction in incidence in dromedary camel populations of 2 million (comparable to that of KSA) made up of 25 homogenous patches of 80,000. Panels show results for different transmission intensities spanning estimates from age-stratified seroprevalence data, and under different vaccine efficacy scenarios. Transparent ribbons show the 2.5-97.5% quantiles across n = 1000 stochastic model runs.*

Table S5. *The vaccine coverage needed to interrupt transmission under the 3 different efficacy scenarios when infectiousness is assumed to be proportional to viral load. Dashes indicate that no level of coverage was sufficient to interrupt transmission. Scenario 1 = the vaccine reduces subsequent infectiousness for all vaccinated animals but does not reduce susceptibility to infection. Scenario 2 = the vaccine reduces both infectiousness and susceptibility of all vaccinated animals. Scenario 3 = the vaccine reduces both infectiousness and susceptibility but only in previously infected animals.*

R_0	1/ρ	Vaccine coverage (%) needed to interrupt transmission in a population of:					
		**75,000** *split into patches of 3,000*			**2,000,000** *split into patches of 80,000*		
		Scenario 1	Scenario 2	Scenario 3	Scenario 1	Scenario 2	Scenario 3
3.5	1	*70*	*70*	-	*40*	*40*	-
	3	*90*	*90*	-	*40*	*40*	-
	10	-	-	-	*40*	*40*	-
7.0	1	*90*	*90*	-	*60*	*60*	-
	3	*100*	*100*	-	*60*	*60*	-
	10	-	-	-	*80*	*80*	-
14.0	1	*100*	*100*	-	*60*	*60*	-
	3	-	-	-	*70*	*70*	-
	10	-	-	-	*80*	*80*	-

Table S6. *The vaccine coverage needed to interrupt transmission under the 3 different efficacy scenarios when infectiousness is assumed to be proportional to the logarithm of the viral load. Dashes indicate that no level of coverage was sufficient to interrupt transmission. Scenario 1 = the vaccine reduces subsequent infectiousness for all vaccinated animals but does not reduce susceptibility to infection. Scenario 2 = the vaccine reduces both infectiousness and susceptibility of all vaccinated animals. Scenario 3 = the vaccine reduces both infectiousness and susceptibility but only in previously infected animals.*

R_0	1/ρ	Vaccine coverage (%) needed to interrupt transmission in a population of:					
		**75,000** *split into patches of 3,000*			**2,000,000** *split into patches of 80,000*		
		Scenario 1	Scenario 2	Scenario 3	Scenario 1	Scenario 2	Scenario 3
3.5	1	-	*90*	-	*60*	*50*	-
	3	-	-	-	*80*	*70*	-
	10	-	-	-	-	-	-
7.0	1	-	-	-	-	*100*	-
	3	-	-	-	-	-	-
	10	-	-	-	-	-	-
14.0	1	-	-	-	-	-	-
	3	-	-	-	-	-	-
	10	-	-	-	-	-	-

References

1. Dighe, A., Jombart, T., Van Kerkhove, M. D. & Ferguson, N. A systematic review of MERS-CoV seroprevalence and RNA prevalence in dromedary camels: Implications for animal vaccination. *Epidemics* **29,** 100350 (2019).
2. Kasem, S., Qasim, I., Al-Hufofi, A., Hashim, O., Alkarar, A., Abu-Obeida, A., Gaafer, A., Elfadil, A., Zaki, A., Al-Romaihi, A., Babekr, N., El-Harby, N., Hussien, R., AL-Sahaf, A., Al-Doweriej, A., Bayoumi, F., Poon, L. L. M., Chu, D. K. W., Peiris, M. & Perera, R. A. P. M. Cross-sectional study of MERS-CoV-specific RNA and antibodies in animals that have had contact with MERS patients in Saudi Arabia. *J. Infect. Public Health* **11,** 331–338 (2018).
3. Wernery, U., El Rasoul, Ih., Wong, E. Y. M., Joseph, M., Chen, Y., Jose, S., Tsang, A. K. L., Patteril, N. A. G., Chen, H., Elizabeth, S. K., Yuen, K. Y., Joseph, S., Xia, N., Wernery, R., Lau, S. K. P. & Woo, P. C. A phylogenetically distinct Middle East respiratory syndrome coronavirus detected in a dromedary calf from a closed dairy herd in Dubai with rising seroprevalence with age. *Emerg. Microbes Infect.* **4,** (2015).
4. Ali, M. A., El-Shesheny, R., Kandeil, A., Shehata, M., Elsokary, B., Gomaa, M., Hassan, N., El Sayed, A., El-Taweel, A., Sobhy, H., Fasina, F. O., Dauphin, G., El Masry, I., Wolde, A. W., Daszak, P., Miller, M., VonDobschuetz, S., Gardner, E., Morzaria, S., Lubroth, J. & Makonnen, Y. J. Cross-sectional surveillance of middle east respiratory syndrome coronavirus (MERS-CoV) in dromedary camels and other mammals in Egypt, August 2015 to January 2016. *Eurosurveillance* **22,** (2017).
5. Ali, M. A., Shehata, M. M., Gomaa, M. R., Kandeil, A., El-Shesheny, R., Kayed, A. S., El-Taweel, A. N., Atea, M., Hassan, N., Bagato, O., Moatasim, Y., Mahmoud, S. H., Kutkat, O., Maatouq, A. M., Osman, A., McKenzie, P. P., Webby, R. J. & Kayali, G. Systematic, active surveillance for Middle East respiratory syndrome coronavirus in camels in Egypt. *Emerg. Microbes Infect.* **6,** (2017).
6. Kandeil, Gomaa, Nageh, Shehata, Kayed, Sabir, Abiadh, Jrijer, Amr, Said, Byarugaba, Wabwire-Mangen, Tugume, Mohamed, Attar, Hassan, Linjawi, Moatassim, Kutkat, Mahmoud, Bagato, Shama, El-Shesheny, Mostafa, Perera, Chu, Hassan, Elsokary, Saad, Sobhy, El Masry, McKenzie, Webby, Peiris, Makonnen, Ali, & Kayali. Middle East Respiratory Syndrome Coronavirus (MERS-CoV) in Dromedary Camels in Africa and Middle East. *Viruses* **11,** 717 (2019).

7. Hens, N., Aerts, M., Faes, C., Shkedy, Z., Lejeune, O., Damme, P. V. & Beutels, P. Seventy-five years of estimating the force of infection from current status data. *Epidemiol. Infect.* **138,** 802–812 (2010).

8. Imai, N., Dorigatti, I., Cauchemez, S. & Ferguson, N. M. Estimating Dengue Transmission Intensity from Case-Notification Data from Multiple Countries. *PLoS Negl. Trop. Dis.* **10,** e0004833 (2016).

9. Reusken, C. B. E. M., Messadi, L., Feyisa, A., Ularamu, H., Godeke, G. J., Danmarwa, A., Dawo, F., Jemli, M., Melaku, S., Shamaki, D., Woma, Y., Wungak, Y., Gebremedhin, E. Z., Zutt, I., Bosch, B. J., Haagmans, B. L. & Koopmans, M. P. G. Geographic distribution of MERS coronavirus among dromedary camels, Africa. *Emerg. Infect. Dis.* **20,** 1370–1374 (2014).

10. Deem, S. L., Fèvre, E. M., Kinnaird, M., Browne, A. S., Muloi, D., Godeke, G. J., Koopmans, M. & Reusken, C. B. Serological evidence of MERS-CoV antibodies in dromedary camels (camelus dromedaries) in laikipia county, Kenya. *PLoS ONE* **10,** (2015).

11. Munyua, P., Corman, V. M., Bitek, A., Osoro, E., Meyer, B., Müller, M. A., Lattwein, E., Thumbi, S. M., Murithi, R., Widdowson, M. A., Drosten, C. & Njenga, M. K. No serologic evidence of middle east respiratory syndrome coronavirus infection among camel farmers exposed to highly seropositive camel herds: A household linked study, Kenya, 2013. *Am. J. Trop. Med. Hyg.* **96,** 1318–1324 (2017).

12. Ommeh, S., Zhang, W., Zohaib, A., Chen, J., Zhang, H., Hu, B., Ge, X. Y., Yang, X. L., Masika, M., Obanda, V., Luo, Y., Li, S., Waruhiu, C., Li, B., Zhu, Y., Ouma, D., Odendo, V., Wang, L. F., Anderson, D. E., Lichoti, J., Mungube, E., Gakuya, F., Zhou, P., Ngeiywa, K. J., Yan, B., Agwanda, B. & Shi, Z. L. Genetic Evidence of Middle East Respiratory Syndrome Coronavirus (MERS-Cov) and Widespread Seroprevalence among Camels in Kenya. *Virol. Sin.* **33,** 484–492 (2018).

13. Thwiny, H. T., Al Hamed, T. A. & Nazzal, A. R. Seroepidemiological study of Middle East respiratory syndrome (MERS) virus infection in Iraqi dromedary camels. *Vet. Arh.* **88,** 191–200 (2018).

14. Van Doremalen, N., Hijazeen, Z. S. K., Holloway, P., Al Omari, B., Mcdowell, C., Adney, D., Talafha, H. A., Guitian, J., Steel, J., Amarin, N., Tibbo, M., Abu-Basha, E., Al-Majali, A. M., Munster, V. J. & Richt, J. A. High prevalence of middle east respiratory coronavirus in young dromedary camels in Jordan. *Vector-Borne Zoonotic Dis.* **17,** 155–159 (2017).

15. Alagaili, A. N., Briese, T., Mishra, N., Kapoor, V., Sameroff, S. C., de Wit, E., Munster, V. J., Hensley, L. E., Zalmout, I. S., Kapoor, A., Epstein, J. H., Karesh, W. B., Daszak, P., Mohammed, O. B. & Ian Lipkin, W.

Middle east respiratory syndrome coronavirus infection in dromedary camels in Saudi Arabia. *mBio* **5,** (2014).

16. Harrath, R. & Abu Duhier, F. M. Sero-prevalence of Middle East respiratory syndrome coronavirus (MERS-CoV) specific antibodies in dromedary camels in Tabuk, Saudi Arabia. *J. Med. Virol.* **90,** 1285–1289 (2018).
17. Hemida, M. G., Perera, R. A., Wang, P., Alhammadi, M. A., Siu, L. Y., Li, M., Poon, L. L., Saif, L., Alnaeem, A. & Peiris, M. Middle east respiratory syndrome (MERS) coronavirus seroprevalence in domestic livestock in Saudi Arabia, 2010 to 2013. *Eurosurveillance* **18,** (2013).
18. Wernery, U., Corman, V. M., Wong, E. Y. M., Tsang, A. K. L., Muth, D., Lau, S. K. P., Khazanehdari, K., Zirkel, F., Ali, M., Nagy, P., Juhasz, J., Wernery, R., Joseph, S., Syriac, G., Elizabeth, S. K., Patteril, N. A. G., Woo, P. C. Y. & Drosten, C. Acute middle east respiratory syndrome coronavirus infection in livestock dromedaries, Dubai, 2014. *Emerg. Infect. Dis.* **21,** 1019–1022 (2015).
19. Islam, A., Epstein, J. H., Rostal, M. K., Islam, S., Rahman, M. Z., Hossain, M. E., Uzzaman, M. S., Munster, V. J., Peiris, M., Flora, M. S., Rahman, M. & Daszak, P. Middle east respiratory syndrome Coronavirus antibodies in dromedary camels, Bangladesh, 2015. *Emerg. Infect. Dis.* **24,** 926–928 (2018).
20. Saqib, M., Sieberg, A., Hussain, M. H., Mansoor, M. K., Zohaib, A., Lattwein, E., Müller, M. A., Drosten, C. & Corman, V. M. Serologic evidence for MERS-CoV infection in Dromedary Camels, Punjab, Pakistan, 2012–2015. *Emerg. Infect. Dis.* **23,** 550–551 (2017).
21. Zohaib, A., Saqib, M., Athar, M. A., Chen, J., Sial, A. ur R., Khan, S., Taj, Z., Sadia, H., Tahir, U., Tayyab, M. H., Qureshi, M. A., Mansoor, M. K., Naeem, M. A., Hu, B. J., Khan, B. A., Ujjan, I. D., Li, B., Zhang, W., Luo, Y., Zhu, Y., Waruhiu, C., Khan, I., Yang, X. L., Sajid, M. S., Corman, V. M., Yan, B. & Shi, Z. L. Countrywide Survey for MERS-Coronavirus Antibodies in Dromedaries and Humans in Pakistan. *Virol. Sin.* **33,** 410–417 (2018).
