# Supplementary material for: Modelling transmission of Middle East respiratory syndrome coronavirus in camel populations and the potential impact of animal vaccination
Source: Nat Commun. 2025 Aug 18;16:7679. doi: 10.1038/s41467-025-62365-x (PMC12361541; doi:10.1038/s41467-025-62365-x)
Supplement: Supplementary file 2 — Reporting Summary [file 41467_2025_62365_MOESM2_ESM.pdf]

nature portfolio

Corresponding author(s): Amy Dighe

Last updated by author(s): Jul 11, 2025

Reporting Summary

Nature Portfolio wishes to improve the reproducibility of the work that we publish. This form provides structure for consistency and transparency in reporting. For further information on Nature Portfolio policies, see our Editorial Policies and the Editorial Policy Checklist.

Statistics

For all statistical analyses, confirm that the following items are present in the figure legend, table legend, main text, or Methods section.

n/a	Confirmed	
☐	☒	The exact sample size (*n*) for each experimental group/condition, given as a discrete number and unit of measurement
☐	☒	A statement on whether measurements were taken from distinct samples or whether the same sample was measured repeatedly
☐	☒	The statistical test(s) used AND whether they are one- or two-sided<br>*Only common tests should be described solely by name; describe more complex techniques in the Methods section.*
☐	☒	A description of all covariates tested
☐	☒	A description of any assumptions or corrections, such as tests of normality and adjustment for multiple comparisons
☐	☒	A full description of the statistical parameters including central tendency (e.g. means) or other basic estimates (e.g. regression coefficient) AND variation (e.g. standard deviation) or associated estimates of uncertainty (e.g. confidence intervals)
☒	☐	For null hypothesis testing, the test statistic (e.g. *F*, *t*, *r*) with confidence intervals, effect sizes, degrees of freedom and *P* value noted<br>*Give P values as exact values whenever suitable.*
☐	☒	For Bayesian analysis, information on the choice of priors and Markov chain Monte Carlo settings
☐	☒	For hierarchical and complex designs, identification of the appropriate level for tests and full reporting of outcomes
☐	☒	Estimates of effect sizes (e.g. Cohen's *d*, Pearson's *r*), indicating how they were calculated

Our web collection on statistics for biologists contains articles on many of the points above.

Software and code

Policy information about availability of computer code

Data collection

This study was limited to secondary analysis of published data. However, some of the secondary data was extracted from published Figures using:

PlotDigitizer. PlotDigitizer: Extract Data from Graph Image Online. (2022). at <https://plotdigitizer.com/> version 2.2

Data analysis	All models presented in this manuscript and the code used to produce the analyses are available from: Dighe, A. Code to accompany: Modelling transmission of Middle East respiratory syndrome coronavirus in camel populations and the potential impact of animal vaccination. (2025). at <https://doi.org/10.5281/zenodo.15864039>. version 1.0.<br><br>The following existing packages were used:<br><br>The statistical programming Language, R: R Core Team. R: A Language and Environment for Statistical Computing. (2021). at <https://www.r-project.org/> version 3.5.3<br><br>Odin: FitzJohn, R. & Fischer, T. odin: ODE generation and Integration. (2022). at <https://github.com/mrc-ide/odin> version 1.5.11<br><br>Odin.dust: FitzJohn, R. & Lees, J. odin.dust: Compile Odin to Dust. (2022). at <https://github.com/mrc-ide/odin.dust> version 0.3.13<br><br>Stan Development Team. RStan□: the R interface to Stan. R Package (2020). at <https://mc-stan.org/> version 2.32.7

For manuscripts utilizing custom algorithms or software that are central to the research but not yet described in published literature, software must be made available to editors and reviewers. We strongly encourage code deposition in a community repository (e.g. GitHub). See the Nature Portfolio guidelines for submitting code & software for further information.

Data

Policy information about availability of data

All manuscripts must include a data availability statement. This statement should provide the following information, where applicable:

- Accession codes, unique identifiers, or web links for publicly available datasets
- A description of any restrictions on data availability
- For clinical datasets or third party data, please ensure that the statement adheres to our policy

All data used in model fitting or parameterisation are available either within this published article or at https://doi.org/10.5281/zenodo.15864039

Research involving human participants, their data, or biological material

Policy information about studies with human participants or human data. See also policy information about sex, gender (identity/presentation), and sexual orientation and race, ethnicity and racism.

Reporting on sex and gender	NA
Reporting on race, ethnicity, or other socially relevant groupings	NA
Population characteristics	NA
Recruitment	NA
Ethics oversight	NA

Note that full information on the approval of the study protocol must also be provided in the manuscript.

Field-specific reporting

Please select the one below that is the best fit for your research. If you are not sure, read the appropriate sections before making your selection.

☒ Life sciences ☐ Behavioural & social sciences ☐ Ecological, evolutionary & environmental sciences

For a reference copy of the document with all sections, see nature.com/documents/nr-reporting-summary-flat.pdf

Life sciences study design

All studies must disclose on these points even when the disclosure is negative.

Sample size	This was a mathematical modelling study using 23 publicly available secondary data sources gathered in a previous systematic review. We did not perform sample size calculations. In terms of stochastic model runs, during simulations we ran 200-1000 stochastic iterations of each model to ensure representative central estimates - assessed by consistency across runs. During Bayesian model fitting, chains were run for 10000 iterations which was more than necessary to allow convergence and capture a representative sample of the posterior distribution.
Data exclusions	We describe our choice to exclude 3 studies from our analysis as follows: "In total, three studies that were included in the systematic review of MERS-CoV seroprevalence in dromedaries were excluded from our analysis. Two of these studies were excluded as their sampling strategies were not appropriate for FoI estimates which assumes a random cross-sectional sample of seroprevalence. The first sampled dromedaries with epidemiological connection to human cases, and the second sampled camels in response to finding a dromedary which

tested positive for MERS-CoV during what turned out to be the height of an outbreak Finally, the third study was excluded because it was not possible to determine whether the sample population overlapped with another larger study conducted in the same area that was included. To make the geographical range of the FoI estimates as comprehensive as possible, seroprevalence measures from one additional study published after the systematic review were also included. This further study included measures of seroprevalence in camel populations in Senegal and Uganda - two additional countries not represented in the literature previously."

Replication

This study did not involve running experiments in a population or laboratory, rather we used existing published data to infer transmission dynamics of MERS-CoV in camels, and simulation potential impact of vaccination strategies. In terms replication of stochastic model runs, we ran 200-1000 stochastic iterations of each model to ensure representative central estimates - assessed by consistency across runs. In terms of reproducibility, we have insured that our analysis itself is replicable by others through sharing all the code and data used to implement our models and conduct our analysis.

Randomization

Randomisation was not done. This was not an applicable approach to use in our study, which was a mathematical modelling analysis of 23 sets of publicly available seroprevalence estimates, used to infer and simulate transmission dynamics of a pathogen.

Blinding

Blinding was not done. This was not applicable to our study, which was a mathematical modelling analysis of 23 publicly available secondary seroprevalence estimates used to infer and simulate transmission dynamics of a pathogen.

Reporting for specific materials, systems and methods

We require information from authors about some types of materials, experimental systems and methods used in many studies. Here, indicate whether each material, system or method listed is relevant to your study. If you are not sure if a list item applies to your research, read the appropriate section before selecting a response.

Materials & experimental systems

n/a	Involved in the study
☒	☐ Antibodies
☒	☐ Eukaryotic cell lines
☒	☐ Palaeontology and archaeology
☒	☐ Animals and other organisms
☒	☐ Clinical data
☒	☐ Dual use research of concern
☒	☐ Plants

Methods

n/a	Involved in the study
☒	☐ ChIP-seq
☒	☐ Flow cytometry
☒	☐ MRI-based neuroimaging

Plants

Seed stocks

NA

Novel plant genotypes

NA

Authentication

NA
